# Treatment advocate tactics to expand access to antiviral therapy for HIV and viral hepatitis C in low‐ to high‐income settings: making sure no one is left behind

**DOI:** 10.1002/jia2.25060

**Published:** 2018-04-10

**Authors:** Céline Grillon, Priti R Krishtel, Othoman Mellouk, Anton Basenko, James Freeman, Luís Mendão, Isabelle Andrieux‐Meyer, Sébastien Morin

**Affiliations:** ^1^ Médecins du Monde Paris France; ^2^ Initiative for Access, Medicines and Knowledge Oakland, CA USA; ^3^ International Treatment Preparedness Coalition Marrakech Morocco; ^4^ Alliance for Public Health Kiev Ukraine; ^5^ FixHepC Hobart Australia; ^6^ European AIDS Treatment Group Lisbon Portugal; ^7^ Drugs for Neglected Diseases initiative Geneva Switzerland; ^8^ International AIDS Society Geneva Switzerland

**Keywords:** human immunodeficiency virus, hepatitis C virus, co‐infection, access, patent, low‐ and middle‐income countries, direct‐acting antivirals, people who inject drugs

## Abstract

**Introduction:**

Worldwide, 71 million people are infected with hepatitis C virus (HCV), which, without treatment, can lead to liver failure or hepatocellular carcinoma. HCV co‐infection increases liver‐ and AIDS‐related morbidity and mortality among HIV‐positive people, despite ART. A 12‐week course of HCV direct‐acting antivirals (DAAs) usually cures HCV – regardless of HIV status. However, patents and high prices have created access barriers for people living with HCV, especially people who inject drugs (PWID). Inadequate access to and coverage of harm reduction interventions feed the co‐epidemics of HIV and HCV; as a result, the highest prevalence of HCV is found among PWID, who face additional obstacles to treatment (including stigma, discrimination and other structural barriers). The HIV epidemic occurred during globalization of intellectual property rights, and highlighted the relationship between patents and the high prices that prevent access to medicines. Indian generic manufacturers produced affordable generic HIV treatment, enabling global scale‐up. Unlike HIV, donors have yet to step forward to fund HCV programmes, although DAAs can be mass‐produced at a low and sustainable cost. Unfortunately, although voluntary licensing agreements between originators and generic manufacturers enable low‐income (and some lower‐middle income countries) to buy generic versions of HIV and HCV medicines, most middle‐income countries with large burdens of HCV infection and HIV/HCV co‐infection are excluded from these agreements. Our commentary presents tactics from the HIV experience that treatment advocates can use to expand access to DAAs.

**Discussion:**

A number of practical actions can help increase access to DAAs, including new research and development (R&D) paradigms; compassionate use, named‐patient and early access programmes; use of TRIPS flexibilities such as compulsory licences and patent oppositions; and parallel importation via buyers’ clubs. Together, these approaches can increase access to antiviral therapy for people living with HIV and viral hepatitis in low‐, middle‐ and high‐income settings.

**Conclusions:**

The HIV example provides helpful parallels for addressing challenges to expanding access to HCV DAAs. HCV treatment access – and harm reduction – should be massively scaled‐up to meet the needs of PWID, and efforts should be made to tackle stigma and discrimination, and stop criminalization of drug use and possession.

## 1 Introduction

Worldwide, an estimated 71 million people have chronic hepatitis C virus (HCV) infection; 2.3 million of them are HIV co‐infected 1. The highest prevalence of HCV infection – 82% – is found among HIV‐infected people who inject drugs (PWID) 2. HIV co‐infection increases the risk for, and accelerates the rate of hepatitis C disease progression, despite use of antiviral therapy 3. In turn, HCV co‐infection more than doubles the mortality rate among HIV‐positive people 4. Lower survival in HIV/HCV co‐infected PWID is due in part to structural barriers, such as criminalization; mandated drug treatment; 5 homelessness; stigma and discrimination in healthcare settings; lack of HIV education and support; provider concerns about adherence and drug resistance; lack of linkage between HIV treatment programmes and needle/syringe exchange programmes, and competing survival priorities (linked to poverty and marginalization) 6, 7. These factors, and others, have limited HCV treatment access for PWID, such as the historical exclusion of PWID from HCV clinical trials, which has led providers to withhold HCV treatment, due to lack of evidence, fears about poor adherence and concerns about post‐treatment reinfection 8.

Recently, a pair of DAA clinical trials in people who were using and/or injecting drugs during HCV treatment reported adherence and cure rates similar to non‐users 9, 10. Guidelines from the World Health Organization (WHO), the American Association for the Study of Liver Disease (AASLD)/Infectious Diseases Society of America (IDSA), the European Association for the Study of the Liver (EASL), and the International Network for Hepatitis in Substance Users (INHSU) 11, 12, 13, 14 now recommend treatment for PWID. Nonetheless, regardless of their HIV status, PWID are less likely to be treated for HCV than non‐injectors, often through policies that increase discrimination in healthcare by restricting access to DAAs based on recent drug use 15, 16.

DAAs are still too expensive for individual patients and as public health tools. Innovative approaches are needed to address complex regulatory requirements, intellectual property, and licensing agreements to improve access to affordable DAAs.

Our commentary presents tactics drawn from the HIV experience that treatment advocates can use to expand access to DAAs in different settings, and ensure that marginalized populations – including PWID – are not left behind. This is essential if the world is to reach the targets set by the WHO for elimination of hepatitis C as a public health concern by 2030 17.

## 2 Discussion

### 2.1 Access challenges

Affordable generic antiretrovirals (ARVs) for HIV treatment have made it possible to scale‐up HIV treatment access, but geographic barriers and high prices limit access to DAAs. The HCV epidemic is concentrated in middle‐income countries (MICs) 18, which will be home to the majority of HIV‐positive people by 2020 19. However, global donors are reluctant to support HCV programmes, and are reducing HIV funding to these countries 20. For example, the world's highest prevalence of HIV/HCV co‐infection is found in Eastern Europe and Central Asia 2, a region that has been experiencing the deepest Global Fund cuts (which may reach 40%–50% in the coming years) 21.

**Figure 1:**
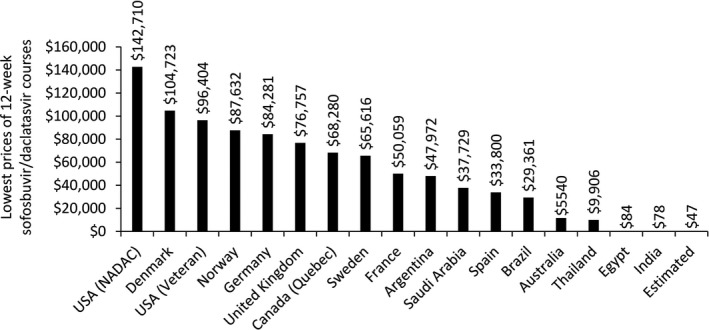
Lowest prices of sofosbuvir/daclatasvir 12‐week courses in selected countries. Estimated: Minimum cost estimation for large‐scale production. Prices are from September 2017 and shown in US$. Used with permission and adapted from Hill 30.

Figure 1 shows some of the latest available pricing figures for sofosbuvir/daclatasvir. The scarcity of published DAA prices makes it difficult to assess price evolutions in different settings. Nevertheless, the available data shows that generic HCV treatment can be produced affordably, and sustainably. A 12‐week course of sofosbuvir and daclatasvir, including profit, could be sold for US $47 22, 30. However, Cipla, Hetero and Mylan in India – the main sources of WHO‐prequalified generic ARVs – and several other generic manufacturers have signed voluntary licenses for sofosbuvir with DAA originator company Gilead Sciences (directly) and for daclatasvir (for which the patent holder is Bristol‐Myers Squibb) with the Medicines Patent Pool. According to a 2003 WHO report on cost‐containment mechanisms for medicines, voluntary licenses allow patent holders to “license to other parties, on an exclusive or nonexclusive basis, the right to manufacture, import, and/or distribute a pharmaceutical product”; they are “usually made for strategic reasons (e.g. market entry) rather than as price gestures and they may not entail any price reduction” 24. Although voluntary licenses improve access to affordable generic medicines in some countries, most MICs are excluded from these agreements (including China, Russia and Turkey, all with more than 500,000 HCV cases 25), which forces them to pay high prices from originator companies. They also often prevent generic manufacturers who sign these agreements from selling to territories outside of the geographic scope of the license – even if a patent is successfully challenged. Although voluntary licenses signed with patent holders directly may not be transparent, voluntary licenses signed through the Medicines Patent Pool are transparent and public‐health‐oriented. For example, the Medicines Patent Pool license with Bristol‐Myers Squibb for daclatasvir states that “generic daclatasvir can be made in any country as long as it is for sale in the countries covered by the agreement” 26. Some generic manufacturers have decided not to sign voluntary licensing agreements (Pharco in Egypt, Beker in Algeria, and Pharma5 in Morocco). Generic DAAs from Pharco and Beker have demonstrated bio‐equivalence 27, and Pharco is expecting WHO prequalification shortly.

MICs may therefore need to pursue several strategies to provide access to DAAs, including use of legal tools to remove patent barriers such as compulsory licenses (a legal mechanism under Trade‐Related Aspects of Intellectual Property Rights, TRIPS, flexibilities that allow governments to produce or import patented medicines without the patent holder's permission, a strategy used by Malaysia in 2017 28) and patent oppositions. Low‐income countries face different access challenges than MICs 29. Although they may be included in voluntary licenses that allow them to purchase generic DAAs, their prices may still be too high, and lack of access to high‐priced HCV diagnostics and limited infrastructure makes it challenging for these countries to bring HCV treatment to scale.

### 2.2 New R&D paradigms to provide accessible medicines

The Drugs for Neglected Diseases *initiative* (DND*i)* has been working with Pharco to develop an affordable, easy‐to‐use, highly efficacious and safe oral pan‐genotypic regimen, for a public health approach, as part of a “test and cure” strategy 30. DND*i* has taken a non‐exclusive license on ravidasvir (an investigational NS5A inhibitor), and intends to make it widely, following successful clinical trials and regulatory approval, available through sub‐licenses to regional or local industrial partners.

### 2.3 Originator access programmes: compassionate use, named‐patient and early access

Compassionate use, named‐patient, and early access programmes are not meant to address public health needs or support elimination campaigns (although they are vital for many individuals). Compassionate use programmes are initiated to serve unmet medical needs while regulators are reviewing dossiers; early access programmes provide medicines during pricing negotiations (e.g. to prevent patients on the verge of life‐threatening liver disease situation from waiting).

Originators should offer compassionate use/early access programmes during negotiations and in countries where they have not sought marketing authorization and where there are no generics available. They should also be encouraged to provide no‐cost access for people with advanced liver disease living in settings with access challenges.

### 2.4 Using patent challenges to ensure access to affordable generic medicines

Patent opposition is the process by which non‐State actors challenge the legality of a patent. Treatment advocates have been opposing patents to secure access to affordable generic medicines, including for HIV drugs (tenofovir disoproxil fumarate's patent was revoked in India in 2009) 32. As recently recommended by the United Nations Secretary‐General's High‐Level Panel on Access to Medicines, countries should make full use of public health safeguards contained in the TRIPS agreement to ensure that patents and other intellectual property restrictions do not prevent access to affordable medicines 23.

Patent opposition is a powerful tool for civil society to oppose undeserved patents and secure access to affordable generic medicines when governments are unwilling or unable to do so. The profusion of patents covering a single medication and the absence of provisions for patent opposition in certain countries may remain challenging for the use of patent opposition as a public health tool. In addition, countries that have introduced additional exclusivity protection (such as data and market exclusivity) face delays in access to affordable generic drugs, even in the absence of patent protection.

Patents covering DAAs have been opposed by the Initiative for Medicines, Access & Knowledge (I‐MAK) and other civil society organizations in Argentina, Brazil, China, India, Russia and Ukraine 33, while HCV treatment rationing in high‐income countries led Médecins du Monde (also known as Doctors of the World) and others to oppose patents on HCV medicines before the European patent office 34. Patent oppositions have already led to the rejection of key patents on sofosbuvir in China and Ukraine 36, and to its substantial weakening in Brazil and Europe 34. Gilead Sciences has appealed the decisions in India and Europe. Key patents on sofosbuvir and daclatasvir have also been rejected by the patent office in Egypt. Oppositions challenging patents covering daclatasvir and velpatasvir are under examination in India 35, 37. Table 1 provides a list of HCV treatment patent oppositions to date.

**Table 1:** HCV DAAs patent oppositions. Adapted and updated from the World Community Advisory Board on HCV Generics and Diagnostics 38

Patent opposed	Patent international publication number	Country or region	National publication number	Opponent (civil society only)	Year	Challenge status
Sofosbuvir (prodrug)	WO2008121634	Argentina		FGEP	2015	Under examination
		China		I‐MAK	2015	Patent rejected in 2015, appeal pending
		Europe	EP2203462	MDM	2015	Maintained in an amended form; under appeal
		India		DNP+, I‐MAK	2013	Under examination
		Russia		ITPCru	2015	Partially revoked (Appeal)
		Thailand		AAF	2016	Under examination
		USA	US7964580	I‐MAK	2017	Filed
		USA	US 8735372	I‐MAK	2017	Filed
		USA	US 8334270	I‐MAK	2017	Filed
Sofosbuvir (base compound/molecule)	WO2005003147	Argentina		FGEP	2017	Opposition filed
		Brazil		ABIA	2015	Opposition filed, preliminary rejection by ANVISA, under examination
		China		I‐MAK	2017	Invalidation filed, case pending
		Europe	EP2604620	MDM	2017	Under examination
		Europe	EP2604620	MSF	2017	Under examination
		Europe	EP2604620	Consortium of six European NGOs	2017	Under examination
		India		DNP+, I‐MAK	2013	Refused first but granted later. In the process of appeal
		USA	US7429572	I‐MAK	2017	Filed
Sofosbuvir (crystalline)	WO2011123645	USA	US8633309	I‐MAK	2017	Filed
		USA	US9284342	I‐MAK	2017	Filed
Sofosbuvir (polymorphs)	WO2011123645	India		DNP+, I‐MAK	2017	Under examination
		Ukraine	a201212444	AUN of PLWH, I‐MAK	2015	Under examination
Sofosbuvir (process)	WO2012012465	Ukraine	a201301999	AUN of PLWH	2016	Rejected
Sofosbuvir/ledipasvir (compound)	WO2013040492 A2	Ukraine	a201403617	AUN of PLWH	2016	Under examination
Daclatasvir (crystalline)	WO2009020828	India		DNP+, I‐MAK	2017	Under examination
Daclatasvir (intermediate)	WO2008021927	India		LC	2017	Under examination
Velpatasvir (base)	WO2013075029	India		DNP+, I‐MAK	2017	Under examination

### 2.5 Parallel importation via buyers’ clubs

Buyers’ clubs leverage the TRIPS flexibility outlined in Article 60 – De Minimus Imports, which states “Members may exclude from the application of the above provisions small quantities of goods of a non‐commercial nature contained in travellers’ personal luggage or sent in small consignments” 39. Most countries allow personal medication importation, including receiving a 3‐month supply of medicine through the mail.

Reputable buyers’ clubs can help patients navigate the unfamiliar and potentially dangerous process of personal importation, operating as an advocate/agent to comply with laws dictating that only pharmacists can sell medications, while the patient remains the legal buyer and importer. Buyers’ clubs provide government and insurers with breathing space and a better negotiating position. In negotiations, delay and volume restriction are the primary tools. Drug companies know that the urgency to get medications to desperately ill patients means that governments and insurers simply cannot hold out indefinitely ‐ and will eventually capitulate to public pressure. In countries where Buyers’ clubs operate effectively (including in Australia, New Zealand, Italy, Switzerland), advantageous price negotiations for DAAs were finalized rapidly.

## 3 Conclusions

The DAA era presents a fantastic opportunity to eliminate HCV – but low‐ and middle‐income countries, and PWID in particular, are being left behind, without access to HCV‐related information, prevention and treatment. Indeed, despite being essential to reach the WHO targets for HCV elimination, expanding access to DAAs will need to be matched by efforts to address the structural barriers faced by PWID: systemic and structural discrimination, stigma and human rights violations 16.

The progress made around access to antiretroviral therapy for HIV provides helpful parallels when confronted with challenges for expanding access to DAAs for HCV treatment. HIV/HCV co‐infection may represent a natural starting point for scaling up HCV treatment coverage, especially for PWID. Although lessons from HIV advocacy give us an understanding of the key pressure points to increase access to HCV antivirals (the DAAs) and eliminate HCV as a public health threat, achieving this victory will be impossible until PWID can access HCV prevention, care and treatment. This is particularly important in Eastern Europe and Central Asia, where there are dramatic donor funding cuts, high HIV/HCV co‐infection rates, and historically repressive drug policies. Drug policy reform, the fulfilment of human rights and the creation of non‐criminalizing environments are critical enablers for any comprehensive attempt to address and reverse the twin epidemics of HIV and HCV among the community of PWID.

DAAs can be mass‐produced at a low and sustainable cost. Unfortunately, although voluntary licensing agreements enable low‐income (and some lower‐middle income countries) to buy generic versions of HIV and HCV medicines, most middle‐income countries with large burdens of HCV and HIV/HCV co‐infection are excluded from these agreements, and therefore face higher prices. A number of practical actions can help increase access to DAAs in low‐, middle‐, and high‐income countries. The tactics presented in our commentary are summarized in Table 2. Together, they can contribute to increasing access to antiviral therapy for HIV and HCV in low‐ to high‐income settings.

**Table 2:** Treatment advocate tactics to expand access to antiviral therapy

Tactics	Pros and cons
New R&D paradigms	+ Potentially very effective globally
	− Depending on large financial resources, high‐level scientific and clinical expertise and subject to a timeline of multiple years
Patent oppositions	+ Potentially very effective at the national or global level
	− Depending on legal expertise and subject to a timeline of multiple years
Advocacy for the use of compulsory licenses	+ Potentially very effective at the national or global level
	− Depending on government action and strong political commitment
Parallel importation via buyers’ clubs	+ Fully legal and relatively simple approach that can increase access to patients locally, while helping countries negotiate lower prices at the national level
	− Limited impact (relatively small number of people directly getting access through this approach)
Originator access programmes	+ Relatively easy to negotiate, taking advantage of drug originators corporate responsibility efforts and importance of public relations
	− Limited impact (relatively small number of people getting access through this approach)

Unfortunately, the repressive laws that criminalize PWID continue to interfere with the treatment and harm reduction programmes that are essential to their health. These programmes are fully effective only when they operate in a supportive legal environment, where PWID know that they will not face police harassment or arrest. Stigma and discrimination within the medical community, including concerns about poor adherence, reinfection, and the lack of treatment settings adapted to the needs of PWID create additional barriers to treatment among PWID. Investment into the development of a medicine called “antistigmavir” may need to accompany expanded access to DAAs.

## Competing interests

PRK, OM, AB, LM and CG declare no competing interest. IAM works for DNDi, which has a partnership agreement with Pharco. JF runs a website called fixhepc.com that assists patients executing a personal medication importation of generic HCV medication. SM works for the International AIDS Society (IAS), which receives funding from a number of different sources, including from the biomedical industry.

## Authors’ contributions

All authors contributed equally to drafting the content, editing and reviewing the manuscript.
